# Mediating effect of health literacy on the relationship between diabetes status and aerobic physical activity adherence among middle-aged Korean adults: a theory-based secondary analysis of the second year of the ninth Korea National Health and Nutrition Examination Survey

**DOI:** 10.7586/jkbns.25.090

**Published:** 2026-05-29

**Authors:** Myoung-Lyun Heo

**Affiliations:** Department of Nursing, Jeonju University, Jeonju, Korea

**Keywords:** Diabetes mellitus, Middle aged, Self care, Health literacy, Exercise

## Abstract

**Purpose:**

This study applied Orem’s self-care theory to examine differences in adherence to aerobic physical activity according to diabetes status among middle-aged adults and to evaluate the mediating effect of health literacy. By distinguishing between diabetes and prediabetes, the study aimed to reflect the stepwise nature of self-care demand while conceptualizing health literacy as a self-care agent.

**Methods:**

A secondary analysis was conducted using data from the second year of the ninth Korea National Health and Nutrition Examination Survey. The study included 2,781 adults aged 40–64 years. Variables were constructed based on Orem’s self-care theory. Complex sample hierarchical logistic regression and mediation analyses were conducted using SPSS version 28.0 and R version 4.4.2.

**Results:**

Among the participants, 14.3% had diabetes and 34.1% had prediabetes. Higher educational attainment and personal income were significantly associated with adherence to aerobic physical activity. Diabetes status was significantly associated with decreased adherence, whereas prediabetes status showed no significant association. Health literacy was positively associated with adherence. Mediation analysis indicated that health literacy partially mediated the negative effects of diabetes and prediabetes on adherence to aerobic physical activity.

**Conclusion:**

By empirically applying Orem’s self-care theory, this study identified differences in adherence to aerobic physical activity according to diabetes status and demonstrated the critical role of health literacy as a self-care agent. These findings suggest that nursing interventions for diabetes prevention and management should adopt stratified approaches based on health status, with an emphasis on enhancing health literacy and strengthening self-care behaviors.

## INTRODUCTION

Middle adulthood, generally defined as the period between 40 and 65 years of age, is a period marked by prominent physical and metabolic changes, such as loss of muscle strength, increased body fat, and heightened insulin resistance, which result from various factors, including a decline in basal metabolic rate, hormonal changes, and reduced physical activity due to aging [1]. These changes lead to the onset of major chronic diseases, and effective health management during middle adulthood becomes a critical factor that influences the quality of life in later years [2].

Among chronic diseases, diabetes is closely associated with individual lifestyle habits, such as diet and physical activity, and its onset can directly lead to a decline in quality of life [3]. In particular, individuals in their 50s and 60s, considered middle-aged, account for more than half of all patients with diabetes [4]. Previous studies based on national data have reported changes in physical activity patterns among Korean adults compared with the pre-pandemic period in 2019, particularly among older age groups [5]. These epidemiological trends suggest that diabetes is not merely a health issue in older adulthood, but a significant public health concern among middle-aged populations, highlighting the importance of early prevention and management efforts during midlife. Furthermore, inadequate prevention and management of diabetes can lead to severe complications such as cardiovascular and kidney diseases [6]. These complications not only reduce individual quality of life but also impose a substantial burden on national healthcare costs [7]. Therefore, prevention and early intervention for diabetes during middle adulthood are key determinants of health status in old age and are essential from a national public health policy perspective [2,8]. Consequently, regardless of whether diabetes has been diagnosed, both preventive and therapeutic management are necessary for middle-aged adults. Preventive management includes lifestyle modifications such as maintaining a healthy diet, engaging in regular physical activity, and monitoring metabolic risk factors, whereas therapeutic management involves the ongoing management of diagnosed conditions through medical treatment, lifestyle regulation, and adherence to recommended health behaviors. Therefore, self-care behaviors constitute a central component of health management during this life stage [9].

Orem’s [10] self-care theory emphasizes the importance of self-care, which refers to the actions that individuals must perform to maintain their health and well-being. This theory explains the need for and role of nursing based on an individual’s self-care capabilities and presents a structured framework involving the interaction among self-care demands, agency, and behaviors, thereby theoretically supporting the necessity of nursing intervention when individuals fail to meet their self-care demands [10,11].

Within this theoretical framework, diabetes represents a chronic condition characterized by high self-care demands, as it is closely associated with daily lifestyle habits such as diet and physical activity. Accordingly, the self-care behaviors required to manage diabetes may vary depending on individuals’ cognitive and functional capacities [12]. Among various self-care behaviors, aerobic physical activity plays a particularly important role, as it positively influences blood glucose regulation [13] and improves insulin sensitivity [14], thereby contributing substantially to the prevention and management of diabetes. However, despite its clinical importance, physical activity levels tend to decline with age among middle-aged adults [15]. This decline has been attributed to multiple internal and environmental barriers, including lack of motivation, limited time, insufficient social support, and existing health conditions [16]. Therefore, the simple provision of health-related knowledge alone is insufficient to promote sustained adherence to physical activity. Instead, nursing interventions that strengthen self-care agent, such as health literacy, are required to promote sustained self-care behaviors, including aerobic physical activity adherence.

Lifestyle behaviors such as regular physical activity, healthy dietary habits, and weight management play an important role in health promotion and the prevention of chronic diseases. Accordingly, growing attention has been paid to factors that facilitate the adoption and maintenance of healthy lifestyle behaviors [17]. However, studies have demonstrated that individuals’ abilities to interpret and implement health information vary depending on their level of understanding [18]. Given these differences, the ability to access, comprehend, evaluate, and utilize health-related information—known as health literacy—has emerged as a critical competency that enables individuals to effectively engage in self-care behaviors [19].

A previous study demonstrated that health literacy significantly influences disease understanding and self-care behaviors among individuals with diabetes [20]. Furthermore, low levels of health literacy were associated with difficulties in blood glucose control and an increased risk of complications [20]. Additionally, a significant correlation has been reported between health literacy and physical activity [21], suggesting that health literacy may play a role in explaining adherence to physical activity. Although previous studies have suggested that low health literacy may contribute to inadequate diabetes prevention and management [20], this study adopted a theory-driven analytical perspective grounded in Orem’s self-care framework. Based on Orem’s self-care theory, disease-related conditions are conceptualized as health deviation self-care requisites that generate new learning requirements and adaptive self-care capacities [10,12]. Accordingly, diabetes status was conceptualized not as a disease outcome variable but as a contextual condition that precedes and shapes changes in self-care agent, including health literacy [10].

Previous studies have reported that individuals with diabetes are less likely to meet recommended levels of physical activity compared with individuals without metabolic disorders [22]. Among various forms of physical activity, aerobic exercise is particularly emphasized in diabetes prevention and management guidelines because it plays a central role in improving glycemic control and metabolic health [13,14]. In addition, glycemic status may influence health literacy, as individuals diagnosed with diabetes are required to acquire disease-related knowledge and develop skills for self-management [23]. Furthermore, health literacy has been identified as an important determinant of health behaviors, including physical activity, because individuals with higher health literacy are more likely to adopt and maintain recommended lifestyle behaviors [24].Therefore, based on Orem’s self-care theory, this study aimed to examine the mediating effect of health literacy, conceptualized as a core component of self-care agent, on the relationship between self-care demands—classified by glycemic status as prediabetes, or diabetes—and the self-care behavior of aerobic physical activity adherence among middle-aged adults. By including middle-aged populations requiring both diabetes prevention and management, this study aims to provide theoretical evidence for understanding the relationships among glycemic status, health literacy, and physical activity within the framework of Orem’s self-care theory and to inform the development of nursing interventions that strengthen health literacy.

## METHODS

### 1. Study design

This study was a theory-based secondary analysis of cross-sectional data from the second year of the ninth Korea National Health and Nutrition Examination Survey (KNHANES). This study aimed to investigate the impact of diabetes status on adherence to aerobic physical activity among middle-aged adults in Korea and to examine the mediating effect of health literacy. This study was reported in accordance with the STROBE (Strengthening the Reporting of Observational Studies in Epidemiology) statement for observational studies.

### 2. Dataset and study participants

This study utilized integrated data from the health interview and health examination surveys of the second year (2023) of the ninth KNHANES, provided by the Korea Disease Control and Prevention Agency (KDCA) [25], because it represents the most recent publicly available dataset at the time of analysis and includes complete information on the variables used in this study. The KNHANES targets the Korean population aged one year and older and employs a stratified two-stage cluster probability sampling design based on the 2019 Population and Housing Census to ensure national representativeness.

In the ninth survey cycle, which covered the years 2022–2024, 576 survey areas and 14,400 households were selected. Each year, 192 areas and approximately 4,800 households were selected for sampling. In each survey area, 25 general households were selected, excluding institutional households, and all individuals aged one year or older in those households were included in the survey. The sampling units were selected from the households’ enumeration districts. The stratification variables included administrative district, urban or rural classification, and housing type. The sample size was further adjusted for sex, age, national area ratio, and household composition.

In this study, adults aged 40–64 years were defined as the middle-aged population [26]. Among the total dataset of 6,929 participants, 2,781 individuals who met the age criteria for middle adulthood were included in the analytic sample. The participant selection process is presented in the Appendix 1. To produce representative population estimates and improve the accuracy of standard error estimation, complex sampling design variables were applied, including stratification (kstrata), clustering (psu), and weighting (w_itex). Participants were not excluded due to missing values in individual variables, and analyses were conducted using all available data.

### 3. Theoretical framework

Orem’s [10] self-care theory is a framework that systematizes the concept of self-care, in which individuals need to maintain health and perform daily functions. Whether or not nursing care is required depends on the balance between an individual’s self-care demands and their ability to perform self-care [10]. This theory consists of three core components: self-care demands, agency, and behaviors. The interactions between these components provide a structural framework for health management.

Diabetes status, health literacy, and adherence to aerobic physical activity were defined as indicators of self-care demands, agency, and behavior. These variables were theoretically mapped to the core components of the self-care framework to examine their conceptual relationships. Self-care demands can be classified into three types: universal, developmental, and health. Diabetes, a chronic disease, is a typical example of health deviation that increases self-care demands [10]. Therefore, this study focused on self-care demands related to deviation, which are heightened by changes in health status [12,27]. Thus, diabetes status was theoretically positioned as a core indicator of health deviation self-care demand within the Orem framework.

According to Orem’s self-care theory, self-care agent is not a static personal attribute, but a dynamic capacity that evolves in response to changing self-care demands [10]. In particular, health deviation conditions such as diabetes increase the complexity of daily self-management tasks and require individuals to acquire new disease-related knowledge, interpret health information, and adapt behavioral strategies [12]. Therefore, in this study, diabetes status was conceptualized as an antecedent contextual condition that generates increased self-care demands and precedes changes in self-care agent, including health literacy, rather than as an outcome variable [10]. This theoretical positioning guided the direction of the mediation model constructed in the present analysis.

Self-care agent refers to the ability to recognize one’s self-care needs and plan and execute actions to meet those needs [12]. In this study, health literacy was conceptualized as the primary cognitive component of self-care agent, reflecting individuals’ capacity to understand, evaluate, and apply health-related information to support self-management behaviors [19].

**Figure 1.**
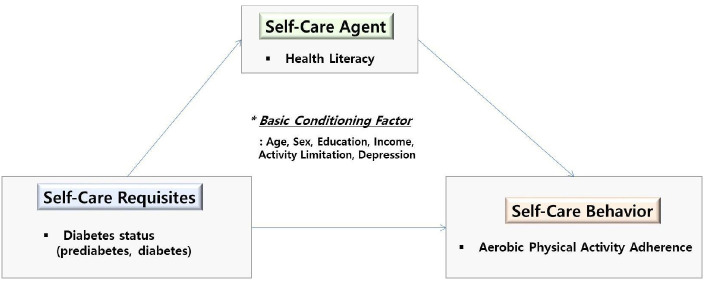
Conceptual framework based on Orem’s self-care theory: relationships between health literacy, diabetes status, and adherence to aerobic physical activity.

Self-care behavior refers to the specific actions taken to fulfill self-care demands. Adherence to aerobic physical activity was used as an indicator in this study. Aerobic physical activity is a representative health behavior that supports health maintenance and the prevention of chronic diseases [27]. Therefore, aerobic physical activity adherence was selected as a representative behavioral indicator of self-care practice within the self-care framework. The theoretical structures of these variables are illustrated in Figure 1.

### 4. Research instruments

This study defined and measured the following variables based on the components of the self-care theory.

#### 1) Self-care demand: diabetes status

Diabetes status was categorized into three groups—normal, prediabetes, and diabetes—based on diagnostic criteria using fasting blood glucose and glycated hemoglobin (HbA1c) values obtained from the health examination data of the second year (2023) of the ninth KNHANES [25].

The normal group included individuals with fasting blood glucose levels < 100 mg/dL, glycated hemoglobin (HbA1c) levels < 5.7%, and no history of diabetes diagnosis or treatment. The prediabetes group included individuals with fasting blood glucose levels between 100 and 125 mg/dL, or HbA1c levels between 5.7% and 6.4%. The diabetes group included individuals with a fasting blood glucose level of 126 mg/dL or higher, an HbA1c level of 6.5% or higher, a physician’s diagnosis of diabetes, or current use of antidiabetic medications or insulin therapy [25].

#### 2) Self-care agent: health literacy

Health literacy refers to the cognitive ability to independently search for, comprehend, evaluate, and apply health-related information to engage in health-management behaviors [19]. In this study, health literacy was measured using the “KNHANES Health Literacy Measurement Tool,” which was developed for monitoring performance indicators under the Health Plan 2030, the Fifth National Health Promotion Plan of Korea [28].

This tool consists of ten items and is structured around four content domains: disease prevention, health promotion, health management, and resource utilization. Each domain consisted of four dimensions: access, understanding, processing/judgment, and application. Responses were measured on a 4-point Likert scale: 1 = not at all true, 2 = not true, 3 = true, and 4 = very true.

During development, the tool demonstrated good internal consistency, with a Cronbach’s alpha of 0.87. In this study, the reliability was not recalculated because of the use of a complex sampling design.

#### 3) Self-care behavior: aerobic physical activity adherence

Adherence to aerobic physical activity was assessed using the variable *pa_aerobic* from the health interview component of the KNHANES. Participants were classified according to whether they met the National Guidelines for Physical Activity.

Individuals were categorized into the adherence group if they engaged in at least 150 minutes of moderate-intensity physical activity per week, at least 75 minutes of vigorous-intensity activity per week, or an equivalent combination of both. Those who did not meet these criteria were classified into the non-adherence group [25].

#### 4) Control variables

To enhance the accuracy and validity of the analysis, sociodemographic and health-related variables that have been reported to influence physical activity adherence and self-care behaviors among middle-aged adults were included as control variables [15,28]. These variables included age, sex, educational level, household income, physical activity limitations, and depressive mood experience.

Age was treated as a continuous variable, whereas sex was categorized as male or female. Educational level was reclassified into two groups: those with a high school diploma or lower and those currently enrolled in college or with higher education. Household income was divided into higher/middle–high and middle–low/lower-income groups. Physical activity limitations were defined as the presence of self-reported functional or health-related restrictions that hinder engagement in physical activities, and were coded as a binary variable (yes/no). Depressive mood was assessed based on its presence or absence [25].

### 5. Data analysis

Data analyses were performed using both SPSS Statistics version 28.0 (IBM Corp., Armonk, NY, USA) and R version 4.4.2 (R Core Team, Vienna, Austria). Descriptive statistics, cross-tabulations, and logistic regression analyses were conducted in SPSS while accounting for the complex sampling design of the KNHANES by incorporating stratification (kstrata), clustering (psu), and sampling weights (w_itex). For categorical variables, unweighted frequencies and weighted percentages were reported, whereas weighted means and standard errors were calculated for continuous variables to reflect the complex survey design.

However, because SPSS does not support mediation analyses that incorporate complex sampling structures, the survey package in R was used to construct the mediation model with complex sample adjustments. Indirect effects were estimated using survey-weighted regression models. Missing values were not excluded from the analytic dataset, and analyses were conducted using all available data. The significance and effect size of each path were visualized using forest plots generated with the forestplot R package to enhance interpretability.

### 6. Ethical considerations

This study used data from the KNHANES, which was approved by the Institutional Review Board (IRB) of the KDCA (approval number: 2022-11-16-R-A). To ensure the protection of participants’ personal information, variables used in the analysis had undergone top coding, bottom coding, and recategorization to prevent individual identification.

Additionally, before conducting the study, the research protocol was reviewed by the IRB of the researcher’s affiliated institution, and an exemption from IRB review was granted (approval number: jjIRB-250529-HR-2025-0501).

## RESULTS

### 1. Differences in aerobic physical activity adherence according to participant characteristics

Using a complex sampling design, 2,781 middle-aged adults were analyzed, and the results are presented in Table 1. Among the participants, 51.6% were classified as having normal glycemic status, 34.1% as having prediabetes and 14.3% as having diabetes. The weighted mean health literacy score was 30.09. The gender distribution was nearly equal, with 50.4% of the population being men and 49.6% being women.

Concerning education, 50.7% had a high school education or lower, and 49.3% had attended college or higher. Household income was evenly distributed, with 49.6% in the low/middle–low group and 50.4% in the high/middle–high group. Most participants (93.6%) reported no physical activity limitations, whereas 6.4% reported limitations. Concerning depressive mood, 9.4% reported having experienced it and 90.6% did not. The mean weighted age of the participants was 52.17 years.

Significant differences in aerobic physical activity adherence were found according to diabetes status (F = 33.09, *p* < .001), health literacy (F = 16.19, *p* < .001), sex (F = 10.53, *p* = .001), educational level (F = 91.79, *p* < .001), household income (F = 10.84, *p* = .001), and physical activity limitation (F = 24.42, *p* < .001).

### 2. Factors influencing aerobic physical activity adherence among middle-aged adults in Korea

Prior to conducting the hierarchical logistic regression analysis, multicollinearity among independent variables was assessed using variance inflation factors (VIF) based on a conventional regression model, and all VIF values were below 10, indicating no serious multicollinearity. Model improvement across hierarchical models was evaluated using likelihood ratio tests. Factors influencing adherence to aerobic physical activity were analyzed using hierarchical logistic regression analysis, and the results are presented in Table 2.

Model 1 included control variables that exhibited statistically significant differences in the chi-square test (sex, education level, household income, and physical activity limitations). Adherence to aerobic physical activity was significantly higher among those with a college education or higher compared to those with a high school education or less (odds ratio [OR] = 1.63, 95% confidence interval [CI] =1.37-1.93, *p* < .001), and among those with high/middle–high household income compared to those with low or middle–low income (OR = 1.24, 95% CI =1.03-1.48, *p* = .023). However, sex and physical activity limitation were not statistically significant.

Model 2 included an independent variable for the diabetes status. Participants with diabetes exhibited significantly lower adherence to aerobic physical activity than those with normal or pre-diabetic status (OR = 0.71, 95% CI =0.56-0.90, *p* = .005), whereas participants with pre-diabetic status did not exhibit statistically significant differences compared to those with normal or diabetic status (OR = 0.98, 95% CI =0.82-1.18, *p* = .831). Educational level (OR = 1.62, 95% CI =1.36-1.93, *p* < .001) and household income (OR = 1.25, 95% CI =1.03-1.50, *p* = .021) remained significant, whereas sex and physical activity limitation remained non-significant.

Model 3 included the mediating variable, health literacy. Health literacy showed a statistically significant association with adherence to aerobic physical activity (OR = 1.02, 95% CI = 1.00–1.04, *p* = .041). Educational level (OR = 1.57, 95% CI = 1.31–1.87, *p* < .001) and household income (OR = 1.23, 95% CI = 1.02–1.48, *p* = .032) remained significantly associated with adherence to aerobic physical activity. Participants with diabetes showed lower odds of adherence compared with those without diabetes (OR = 0.72, 95% CI = 0.56–0.92, *p* = .008). However, sex, physical activity limitations, and prediabetes status were not significantly associated with adherence to aerobic physical activity.

Model fit statistics indicated that the explanatory power of the models slightly increased with the addition of variables. The Nagelkerke R² values were .03 for Model 1, .03 for Model 2, and .03 for Model 3. In addition, the overall Wald F tests indicated that all three models were statistically significant (Model 1: F = 11.76, *p* < .001; Model 2: F = 10.32, *p* < .001; Model 3: F = 8.70, *p* < .001).

### 3. Mediating effect of health literacy on aerobic physical activity adherence in individuals with prediabetes and diabetes

Based on the logistic regression results indicating differences in adherence to aerobic physical activity according to glycemic status, separate mediation analyses were conducted for the prediabetes and diabetes groups. The results are summarized in Table 3.

To control for potential confounding factors, both models included educational level and household income as covariates.

In the analysis of the mediating effect of health literacy on the relationship between prediabetes and aerobic physical activity adherence, prediabetes status had a significant negative effect on health literacy (B = −0.49, *p* = .003), and health literacy had a significant positive effect on aerobic physical activity adherence (B = 0.01, *p* < .001). The direct effect of prediabetes on adherence to aerobic physical activity was also statistically significant (B = −0.04, *p* = .020), indicating that health literacy partially mediated this relationship after adjusting for educational level and household income.

In the diabetes group, diabetes status significantly negatively influenced health literacy (B = −1.27, *p* < .001), and health literacy was positively associated with adherence to aerobic physical activity (B = 0.01, *p* < .001). The direct effect of diabetes on adherence to aerobic physical activity was also significant (B = −0.11, *p* < .001), confirming the partial mediating effect of health literacy, even after controlling for educational level and income.

**Figure 2.**
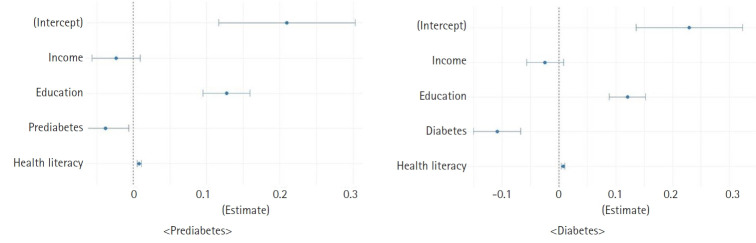
Forest plot of mediation models for adherence to aerobic physical activity: prediabetes and diabetes groups.

To visualize these associations, a forest plot analysis was conducted, as illustrated in Figure 2. In the plot, each dot represents the regression coefficient (estimate), and the horizontal line represents the 95% CI. The results were considered statistically significant if the CI did not include zero. Dots to the right of the reference line indicate positive effects, whereas those to the left indicate negative effects. The analysis revealed that in the prediabetes and diabetes groups, educational level, household income, and health literacy exhibited significant positive associations with adherence to aerobic physical activity, whereas diabetes had a negative effect on adherence.

## DISCUSSION

This study aimed to investigate the relationship between diabetes status and adherence to aerobic physical activity among middle-aged adults in Korea based on Orem’s self-care theory, and to verify the mediating effect of health literacy. The frequency analysis showed that 48.4% of middle-aged adults were classified as having prediabetes or diabetes, indicating that nearly half of this population falls into the at-risk group for diabetes. These findings emphasize the relevance of diabetes prevention and management during midlife and underscore the importance of improving health behaviors through the enhancement of self-care capabilities.

In this study, diabetes was not treated merely as a health outcome indicator, but was conceptualized as an indicator of self-care demands based on Orem’s self-care theory and was set as an independent variable. The association between diabetes and aerobic physical activity—a representative self-care behavior—was analyzed. Among the sociodemographic characteristics that exhibited significant differences in the chi-square analysis, sex, educational level, household income, and physical activity limitations were included as control variables. A complex sample hierarchical logistic regression analysis was performed. Model 1 confirmed that educational level and household income function as important socioeconomic contextual factors influencing adherence to aerobic physical activity, which is consistent with previous domestic and international findings [28,29]. These variables were therefore retained in subsequent models to control for background conditions affecting self-care behavior. Model 2 demonstrated that diabetes, but not prediabetes, was associated with reduced adherence to aerobic physical activity. From the perspective of chronic disease management, psychological burden, reduced self-efficacy, and disease-related physical limitations may constrain individuals’ ability to maintain regular physical activity [27]. These factors may suppress motivation and hinder the translation of self-care intentions into sustained behavioral practice. The observed differences between prediabetes and diabetes indicate that self-care behavior patterns vary according to disease stage and that diabetes substantially increases self-care demands, highlighting the need for targeted nursing interventions to support effective behavioral performance. Model 3 further demonstrated that health literacy plays a significant role in promoting adherence to aerobic physical activity, while educational level, household income, and diabetes status remained significant predictors. These findings suggest that health literacy functions as a core competency that enables individuals to interpret health-related information accurately and translate it into practical self-care behavior [30,31]. Although previous studies have suggested a potential bidirectional relationship between health literacy and diabetes-related outcomes, the present study, grounded in Orem’s self-care theory, interpreted health literacy as a cognitive capacity that is shaped and activated in response to increasing self-care demands during chronic disease management [10,12].

Orem [10] defined self-care performance as a complex and learned ability, indicating that self-care competence encompasses knowledge acquisition, cognitive judgment, and behavioral execution [32]. This suggests that self-care cannot be achieved solely through knowledge delivery, but rather requires the ability to understand and apply information in a practical context. Therefore, the findings of this study support the significance of cognitive ability as emphasized in Orem’s self-care theory. This study identified a significant difference in the adherence to aerobic physical activity between individuals with prediabetes and those with diabetes. Individuals with prediabetes may lack motivation to engage in health behaviors due to being undiagnosed or having low awareness of their condition; moreover, it has been reported that public health interventions and health education for this group are often insufficient [24]. While patients diagnosed with diabetes are more likely to receive ongoing care and education from healthcare professionals, those in the pre-diabetic stage often do not receive such support. Consequently, differences in access to information, educational opportunities, and the clarity of health messages may arise between the two groups, potentially leading to disparities in self-care behaviors [33]. Therefore, in this study, the diabetes status was conceptualized as a condition of health-deviating self-care demands, as proposed by Orem’s self-care theory. Diabetes and prediabetes were set as independent variables, and an additional mediation analysis was conducted to examine the role of health literacy in its relationship with adherence to aerobic physical activity.

Since complex sample data are constructed using stratified and clustered designs, caution is required when making estimates and interpretations compared to those based on simple random sampling. Particularly, model-based approaches such as structural equation modeling face limitations in application because they are less capable of incorporating weights and standard errors derived from complex designs [34]. Therefore, this study employed the traditional regression coefficient-based mediation analysis procedure proposed by Baron and Kenny [35], which considers a complex sampling structure to indirectly assess the significance of the mediation effect. The results indicated that prediabetes and diabetes had a negative direct effect on adherence to aerobic physical activity and that health literacy significantly increased adherence through a partial mediating effect. While logistic regression analysis indicated that prediabetes did not significantly affect adherence to aerobic physical activity, the mediation model revealed a clearer negative effect of prediabetes when the influence of the cognitive variable of health literacy was considered. This suggests that even when the same variables are used, the interpretation of results can vary depending on the analytical method employed, and that the mediation model allows for the identification of latent influences between variables through indirect pathway analysis [36]. These findings provide evidence that individuals in the pre-diabetic group, despite having lower disease awareness than those with diabetes, can still be influenced in their behavior by cognitive capacities such as health literacy. The discrepancy between the non-significant direct effect of prediabetes and the significant indirect effect observed in the mediation analysis may reflect the unique clinical and behavioral characteristics of the prediabetic stage. Prediabetes is often asymptomatic and may not be perceived as an immediate health threat, which can reduce individuals’ motivation to initiate behavioral change [24]. In this context, cognitive factors such as health literacy may function as a critical activating mechanism that facilitates risk recognition and interpretation of health information [30,37]. From the perspective of Orem’s self-care theory, prediabetes may increase self-care demands without automatically translating into behavioral action unless sufficient self-care agent is mobilized [10,12]. This finding suggests that behavioral change in the prediabetic stage is more likely to occur through cognitive and informational pathways rather than through direct disease-driven motivation.

A growing body of research suggests that health literacy plays a significant role in shaping health behaviors, a finding that is further supported by the results of this study. Given that diabetes often progresses silently or presents only mild symptoms that go unnoticed, early prevention is critical. This study conceptualized aerobic physical activity adherence as a key strategy for the prevention and management of diabetes, and demonstrated that improved health literacy can significantly enhance adherence to this approach. As reported in previous studies, individuals with higher health literacy levels tend to engage more actively in self-care behaviors such as aerobic physical activity [30], which highlights the importance of cognitive competence in translating health knowledge into practice. Therefore, it is imperative to develop nursing interventions that aim to enhance health literacy as a means of promoting preventive behaviors in diabetes care.

Orem’s self-care theory is particularly appropriate for explaining the health behaviors of individuals with chronic diseases who require ongoing self-management, as it posits that self-care demands increase in response to disease conditions. In this study, the theory served as an analytical framework, whereby diabetes status was conceptualized as an indicator of self-care demand, and health literacy was positioned as a key component of self-care agent, functioning as a mediating variable. The theoretical structure and empirical verification of the relationships among diabetes status, health literacy, and adherence to aerobic physical activity highlight the significance of this study. Moreover, by applying a mediation analysis to a large-scale, nationally representative dataset with a complex sampling design, this study provides a meaningful empirical example that may serve as a methodological reference for future research employing similar designs.

However, as aerobic physical activity represents only a part of self-care behavior and health literacy does not fully capture the entirety of self-care agent, this study constitutes a preliminary application of Orem’s self-care theory. Moreover, the reliance on self-reported questionnaires imposes limitations on the depth of measurement, which necessitates caution when generalizing the findings. In addition, although middle-aged adults were defined as individuals aged 40–64 years, heterogeneity within this age range was not examined, which may limit age-specific interpretation. Given that individuals with diabetes and prediabetes differ in self-care demands and patterns of practice, future research should stratify the analysis based on group characteristics and age subgroups and continue to link Orem’s theory with a broader range of variables beyond aerobic physical activity to provide a more comprehensive understanding.

## CONCLUSION

This study applied Orem’s self-care theory to examine adherence to aerobic physical activity among Korean middle-aged adults according to diabetes status. The findings indicate that health literacy functions as a key cognitive component of self-care agent that supports behavioral change in both prediabetic and diabetic populations. In particular, the mediating role of health literacy highlights its importance as a core target for nursing interventions aimed at promoting preventive and self-management behaviors.

These results suggest that nursing strategies should prioritize health literacy enhancement to improve adherence to aerobic physical activity, especially during the prediabetic stage when behavioral change may depend more strongly on cognitive and informational pathways. Aerobic physical activity is closely associated with physiological regulation related to metabolic health, including glucose control and cardiovascular function. Therefore, improving health literacy may contribute to maintaining physiological stability and preventing the progression of chronic diseases. These findings suggest that basic nursing plays a fundamental role in assessing and monitoring physiological responses, as well as in promoting self-care behaviors through patient education and the enhancement of health literacy.

Furthermore, the application of a theory-based analytic framework using nationally representative complex sampling data provides empirical support for integrating self-care theory into population-level chronic disease prevention and management programs. Overall, this study offers practical implications for the development of theory-driven, stage-specific nursing interventions and provides a methodological reference for future research applying self-care theory to large-scale health datasets.

## Figures and Tables

**Table 1.** General Characteristics of Middle-aged Adults and Differences in Adherence to Aerobic Physical Activity (N = 2,781)

Type	Variables	Categories	Total		Non-adherence	Adherence	Adjusted Wald F (*p*)
			Unweighted n	Weighted % or estimated mean±SE	No	Yes	
					Weighted % or estimated mean±SE	Weighted % or estimated mean±SE	
Independent	Diabetes status	Normal	2,464	51.6	46.4	53.6	33.09 (< .001)
		Prediabetes	1,570	34.1	54.9	45.1	
		Diabetes	763	14.3	64.0	36.0	
Mediator	Health literacy			30.09 ± 0.13	29.99 ± 0.16	30.85 ± 0.16	16.19 (< .001)
Control	Sex	Men	2,327	50.4	48.7	51.3	10.53 (.001)
		Women	3,061	49.6	53.4	46.6	
	Education	High school or less	3,159	50.7	57.8	42.2	91.79 (< .001)
		College or higher	2,187	49.3	42.9	57.1	
	Income	High or middle–high	2,717	50.4	48.5	51.5	10.84 (.001)
		Low or middle–low	2,658	49.6	53.7	46.3	
	Activity limitation	No	4,886	93.6	49.9	50.1	24.42 (< .001)
		Yes	462	6.4	63.7	36.3	
	Depression	No	4,765	90.6	50.8	49.2	0.16 (.690)
		Yes	591	9.4	51.8	48.2	
	Age (years)			52.17 ± 0.24	52.38 ± 0.28	52.10 ± 0.28	0.78 (.378)

n = non weighted number; SE = Standard error.

**Table 2.** Factors Influencing Adherence to Aerobic Physical Activity in Middle-aged Adults in Korea

		Adherence to aerobic physical activity					
		Model 1		Model 2		Model 3	
Variables	Categories	Wald F (*p*)	OR (95% CI)	Wald F (*p*)	OR (95% CI)	Wald F (*p*)	OR (95% CI)
Sex	Women	1.90 (.169)	1.12 (0.96–1.34)	0.70 (.404)	1.07 (0.91–1.26)	0.37 (.545)	1.05 (0.89–1.24)
Education	College or higher	30.98 (< .001)	1.63 (1.37–1.93)	28.72 (< .001)	1.62 (1.36–1.93)	24.99 (< .001)	1.57 (1.31–1.87)
Income	High or middle–high	5.18 (.023)	1.24 (1.03–1.48)	5.40 (.021)	1.25 (1.03–1.50)	4.62 (.032)	1.23 (1.02–1.48)
Activity limitation	No	0.42 (.519)	1.11 (0.80–1.54)	0.01 (.946)	1.01 (0.72–1.43)	0.00 (.966)	0.99 (0.70–1.40)
Diabetes status	Prediabetes			0.05 (.831)	0.98 (0.82–1.18)	0.02 (.881)	0.99 (0.82–1.19)
	Diabetes			7.89 (.005)	0.71 (0.56–0.90)	7.03 (.008)	0.72 (0.56–0.92)
Health literacy						4.21 (.041)	1.02 (1.00–1.04)
Nagelkerke R²		.03		.03		.03	
Overall Wald F (*p*)		11.76 (< .001)		10.32 (< .001)		8.70 (< .001)	

Reference groups were not practicing (physical activity); men (sex); high school or less (education); low or middle-low (income); yes (activity limitations); normal and diabetes (prediabetes); and normal and prediabetes (diabetes status). Nagelkerke R² indicates model explanatory power; Overall Wald F (*p*) indicates model significance. OR = Odds ratio; CI = Confidence interval.

**Table 3.** Mediating Effect of Health Literacy on Adherence to Aerobic Physical Activity in Individuals with Prediabetes and Diabetes

Group	a (X→M)	b (M→Y)	ab (Indirect effect)	cp (Direct effect)
	B (*p*)	B (*p*)	B (95% CI)	B (*p*)
Prediabetes	−0.49 (.003)	0.01 (< .001)	−0.004 (−0.007 ~ −0.001)	−0.04 (.020)
Diabetes	−1.27 (<.001)	0.01 (< .001)	−0.010 (−0.016 ~ −0.005)	−0.11 (< .001)

X = Prediabetes / Diabetes; M = Health literacy; Y = Adherence to aerobic physical activity; a = X → M; b = M → Y; ab = indirect effect (a×b); cp = direct effect of X on Y, controlling for M; 95% CI = 95% confidence interval. All regression analyses were adjusted for education level and income using complex sample analysis.

## References

[1] Carvalho EL (2025). Mid-life: a critical window for intervention during aging. Asia-Pacific Journal of Surgical & Experimental Pathology.

[2] Ha EH, Lee YM (2020). Influence of midlife health condition and awareness of successful aging on preparation for old age. Korean Journal of Adult Nursing.

[3] Yoo B (2024). Self-management experiences of diabetic patients in one-person household. The Journal of Korean Diabetes.

[4] Health Insurance Review & Assessment Service (2023). HIRA announces diabetes treatment status on World Diabetes Day [Internet]. https://www.hira.or.kr/bbsDummy.do?pgmid=HIRAA020041000100&brdScnBltNo=4&brdBltNo=11327&pageIndex=1.

[5] Park S, Kim H, Kim S, Rhee S, Woo H, Lim H (2023). National trends in physical activity among adults in South Korea before and during the COVID-19 pandemic, 2009-2021. JAMA Network Open.

[6] Chun KH (2011). Evidence-based management and treatment of high-risk individuals with pre-diabetes. Journal of the Korean Medical Association.

[7] Bommer C, Heesemann E, Sagalova V, Manne-Goehler J, Atun R, Bärnighausen T (2017). The global economic burden of diabetes in adults aged 20–79 years: a cost-of-illness study. The Lancet Diabetes & Endocrinology.

[8] Yoo IS (2015). The study of health care utilization and direct medical cost in the diabetes mellitus client. The Journal of the Convergence on Culture Technology.

[9] Riegel B, Jaarsma T, Strömberg A (2012). A middle-range theory of self-care of chronic illness. Advances in Nursing Science.

[10] Orem DE (1991). Nursing: concepts of practice.

[11] Alligood MR (2022). Nursing theorists and their work.

[12] Sousa VD, Zauszniewski JA (2005). Toward a theory of diabetes self-care management. Journal of Theory Construction & Testing.

[13] Frampton J, Cobbold B, Nozdrin M, Oo HTH, Wilson H, Murphy KG (2021). The effect of a single bout of continuous aerobic exercise on glucose, insulin and glucagon concentrations compared to resting conditions in healthy adults: a systematic review, meta-analysis and meta-regression. Sports Medicine.

[14] Yaribeygi H, Atkin SL, Simental-Mendía LE, Sahebkar A (2019). Molecular mechanisms by which aerobic exercise induces insulin sensitivity. Journal of Cellular Physiology.

[15] Luo J, Lee RYW (2022). Opposing patterns in self-reported and measured physical activity levels in middle-aged adults. European Journal of Ageing.

[16] Stutts WC (2002). Physical activity determinants in adults: perceived benefits, barriers, and self-efficacy. AAOHN Journal.

[17] Ferguson C, Jackson D (2017). Selecting, appraising, recommending and using mobile applications (apps) in nursing. Journal of Clinical Nursing.

[18] Panahi R, Aghaeian N, Pishvaei M, Niknami S (2019). The need to pay attention to differences in health literacy and knowledge in health education interventions. Journal of Research & Health.

[19] Van den Broucke S (2014). Health literacy: a critical concept for public health. Archives of Public Health.

[20] Ghaffari-fam S, Lotfi Y, Daemi A, Babazadeh T, Sarbazi E, Dargahi-Abbasabad G (2020). Impact of health literacy and self-care behaviors on health-related quality of life in Iranians with type 2 diabetes: a cross-sectional study. Health and Quality of Life Outcomes.

[21] Buja A, Rabensteiner A, Sperotto M, Grotto G, Bertoncello C, Cocchio S (2020). Health literacy and physical activity: a systematic review. Journal of Physical Activity and Health.

[22] Colberg S, Sigal R, Fernhall B, Regensteiner J, Blissmer B, Rubin R (2010). Exercise and type 2 diabetes: the American College of Sports Medicine and the American Diabetes Association: joint position statement. Diabetes Care.

[23] Osborn C, Bains S, Egede L (2010). Health literacy, diabetes self-care, and glycemic control in adults with type 2 diabetes. Diabetes Technology & Therapeutics.

[24] Geboers B, de Winter A, Luten K, Jansen C, Reijneveld S (2014). The association of health literacy with physical activity and nutritional behavior in older adults, and its social cognitive mediators. Journal of Health Communication.

[25] Korea Disease Control and Prevention Agency (2024). Guidelines for using raw data of the National Health and Nutrition Survey [Internet]. https://knhanes.kdca.go.kr/knhanes/main.do.

[26] Lachman ME (2004). Development in midlife. Annual Review of Psychology.

[27] Kanaley JA, Colberg SR, Corcoran MH, Malin SK, Rodriguez NR, Crespo CJ (2022). Exercise/physical activity in individuals with type 2 diabetes: a consensus statement from the American College of Sports Medicine. Medicine & Science in Sports & Exercise.

[28] Park ST (2024). The association between physical activity levels and health-related quality of life in Korean elderly: the eighth Korea National Health and Nutrition Examination Survey. The Asian Journal of Kinesiology.

[29] Kari JT, Viinikainen J, Böckerman P, Tammelin TH, Pitkänen N, Lehtimäki T (2020). Education leads to a more physically active lifestyle: evidence based on Mendelian randomization. Scandinavian Journal of Medicine & Science in Sports.

[30] Berkman ND, Sheridan SL, Donahue KE, Halpern DJ, Crotty K (2011). Low health literacy and health outcomes: an updated systematic review. Annals of Internal Medicine.

[31] Ghisi GLdM, Aultman C, Konidis R, Foster E, Tahsinul A, Sandison N (2020). Effectiveness of an education intervention associated with an exercise program in improving disease-related knowledge and health behaviours among diabetes patients. Patient Education and Counseling.

[32] Souza VD (2002). Conceptual analysis of self-care agency. Online Brazilian Journal of Nursing.

[33] Luo H, Chen Z, Bell R, Rafferty AP, Gaskins Little NR, Winterbauer N (2020). Health literacy and health behaviors among adults with prediabetes, 2016 Behavioral Risk Factor Surveillance System. Public Health Reports.

[34] Oberski D (2014). lavaan.survey: an R package for complex survey analysis of structural equation models. Journal of Statistical Software.

[35] Baron RM, Kenny DA (1986). The moderator–mediator variable distinction in social psychological research: conceptual, strategic, and statistical considerations. Journal of Personality and Social Psychology.

[36] Yay M (2023). A statistical method frequently used in clinical research in recent years: causal mediation analysis. Turkish Journal of Thoracic and Cardiovascular Surgery.

[37] DN do Ó, Goes AR, Raposo JF, Osborn R, Loureiro I (2024). The impact of health literacy on diabetes control. European Journal of Public Health.

